# Mycophenolate Mofetil Use in Severe Myocarditis Complicating Systemic Lupus

**DOI:** 10.7759/cureus.25789

**Published:** 2022-06-09

**Authors:** Abire Allaoui, Amal EL OUARRADI, Rajaa Jabbouri, Abdelhamid Naitelhou

**Affiliations:** 1 Internal Medicine, Mohammed VI University of Health Sciences (UM6SS), Casablanca, MAR; 2 Internal Medicine, Cheikh Khalifa International University Hospital, Casablanca, MAR; 3 Laboratory of Clinical Immunology, Inflammation and Allergy, Faculty of Medicine and Pharmacy of Casablanca, Hassan II University of Casablanca, Casablanca, MAR; 4 Cardiology, Mohammed VI University of Health Sciences (UM6SS), Casablanca, MAR; 5 Cardiology, Mohammed VI International University Hospital, Casablanca, MAR; 6 Internal Medicine, Cheikh khalifa International University Hospital, Casablanca, MAR

**Keywords:** systemic lupus, mycophenolate mofetil, cardiac magnetic resonance imaging, cardiogenic shock, myocarditis

## Abstract

Cardiac involvement represents an increasingly frequent complication in systemic lupus, with pericarditis being the most classic cardiac manifestation. However, the most severe and fatal form seems to be myocarditis. We present the case of a patient with systemic lupus complicated by cardiogenic shock secondary to troponin-negative acute myopericarditis and successfully treated with mycophenolate mofetil and corticosteroid therapy. A 33-year-old woman with no past medical history presented with asthenia and inflammatory arthralgia. She was admitted in June 2021 for acute heart failure. Transthoracic cardiac ultrasound showed dilated cardiomyopathy with global hypokinesis (20-25% of ejection fraction) and right ventricular dysfunction without significant mitral and aortic valve disease. She had raised proBNP (pro-brain natriuretic peptide), low troponin, normochromic normocytic anemia at 10.4 g/dL, positive direct Coombs, lymphopenia at 460/mm^3^, serum creatinine at 23.9 mg/L, and proteinuria/creatininuria 2.48 g/g. Cardiac magnetic resonance imaging (CMR) suggested the diagnosis of myopericarditis. The etiological assessment did not identify an infectious, toxic, or medicinal cause. The clinical picture suggested the possibility of an autoimmune disease. The patient presented with lesions suggestive of cutaneous vasculitis, with oral ulcers with polyarthritis. The autoimmune workup showed anti-nuclear antibodies at 1:1,280, anti-native DNA antibodies at 210 IU/mL (normal < 10 IU/mL), and positive anti-SM Abs. The diagnosis of lupus myopericarditis complicated by cardiogenic shock was made, which was associated with acute renal impairment. The patient was initiated on heart failure medications along with corticosteroids and mycophenolate mofetil. On day 15, the left ventricular ejection fraction improved to 45-50%, with clinical improvement in signs of heart failure and general condition. The existence of myopericarditis without obvious etiology, especially when there are extra-cardiac signs such as skin and joint involvement, should lead us to look for systemic lupus in order to start etiological treatment in addition to cardiac medical treatment. Until now, there is no standard treatment for lupus myocarditis, but the use of mycophenolate mofetil seems to be a promising treatment.

## Introduction

Cardiovascular disease represents an increasingly frequent complication in systemic lupus with a prevalence of 40-50% [1], with its most classic presentation being pericarditis. Other forms are also known for their severity and mortality rate, such as the occurrence of myocarditis, which occurs in 10% of cases in some autopsy series [2]. Postmortem findings from some cases have reported a higher prevalence of 50-60%, indicating a high prevalence of subclinical disease [3,4]. We present here the case of a patient with systemic lupus, which was revealed by cardiogenic shock on troponin-negative acute myopericarditis and successfully treated with mycophenolate mofetil and corticosteroid therapy.

This article will be presented as an e-poster at the 20th European Congress of Internal Medicine (ECIM 2022) taking place in Malaga, Spain, on June 9-11, 2022.

## Case presentation

**Figure 1:**
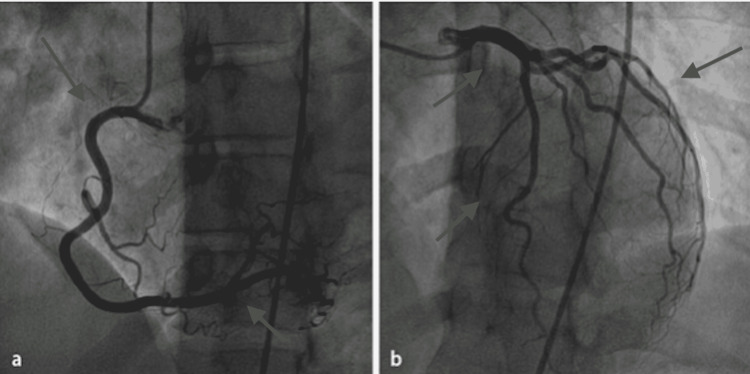
Cardiac catheterization showing normal coronary arteries in our patient. (a) Right coronary artery. (b) Left coronary artery.

A 33-year-old woman who had had asthenia for two years and inflammatory arthralgia of the small joints, which were never investigated, was hospitalized in June 2021 for acute heart failure. On admission to the cardiology intensive care unit, transthoracic echocardiography showed dilated cardiomyopathy with ejection fraction at 20-25% and right ventricular dysfunction without significant mitroaortic valvulopathy. Cardiac catheterization was unremarkable (Figure 1).

**Figure 2:**
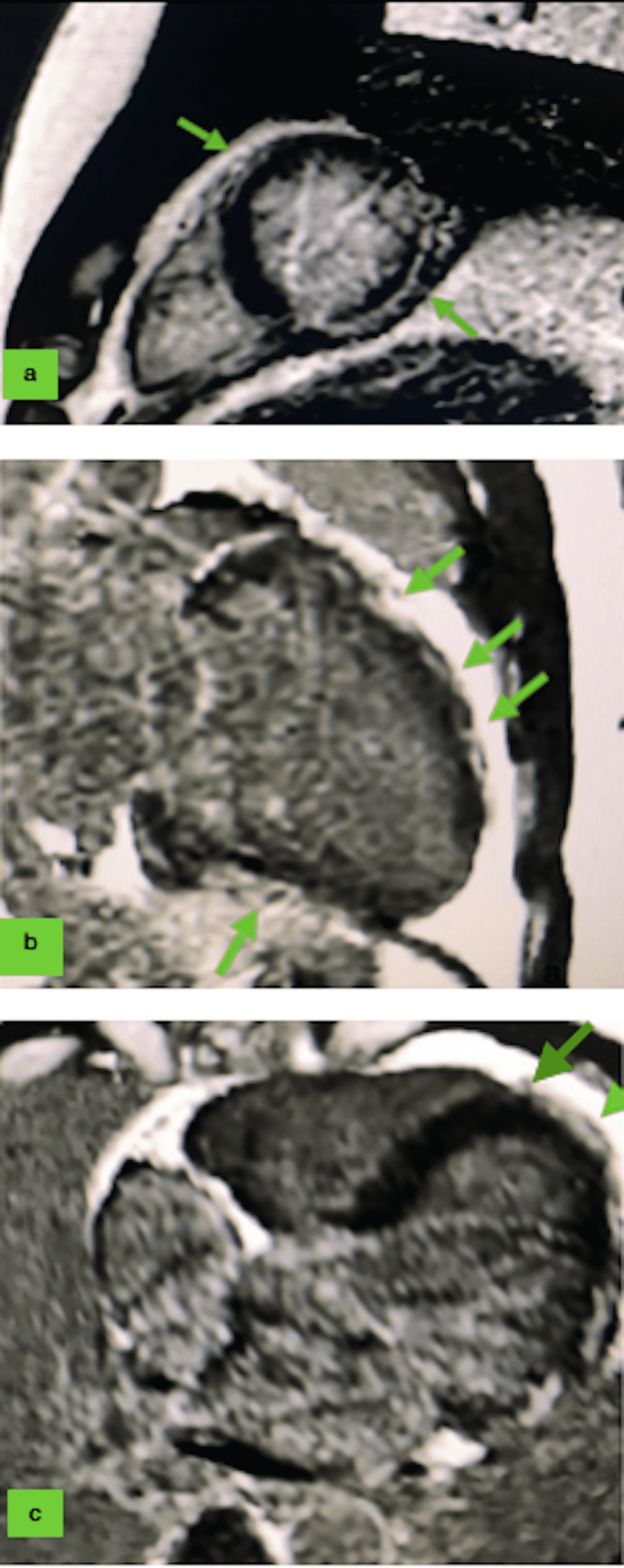
Cardiac MRI of our patient with late gadolinium enhancement showing an epicardial pattern in anterior, inferior, and lateral walls in favor of myocarditis (arrows). (a) Short axis view. (b) Two-chamber view. (c) Three-chamber view.

Laboratory results showed a high proBNP of 54,420 pg/mL (normal < 100pg/mL), a low troponin of 0.03 μg/L (normal < 0.034 μg/L), normochromic normocytic anemia at 10.4 g/dL, positive direct Coombs, lymphopenia at 460/mm^3^, thrombocytopenia at 104 G/L, serum creatinine at 23.9 mg/L, and proteinuria/creatininuria at 2.48 g/g. SARS-CoV-2 serology and PCR testing were negative. EBV, CMV, HVC/HVB, HIV, and influenza testing were also negative. CMR revealed multiple late linear intramyocardial enhancements of both ventricles, suggesting the diagnosis of myocarditis (Figure 2).

**Figure 3:**
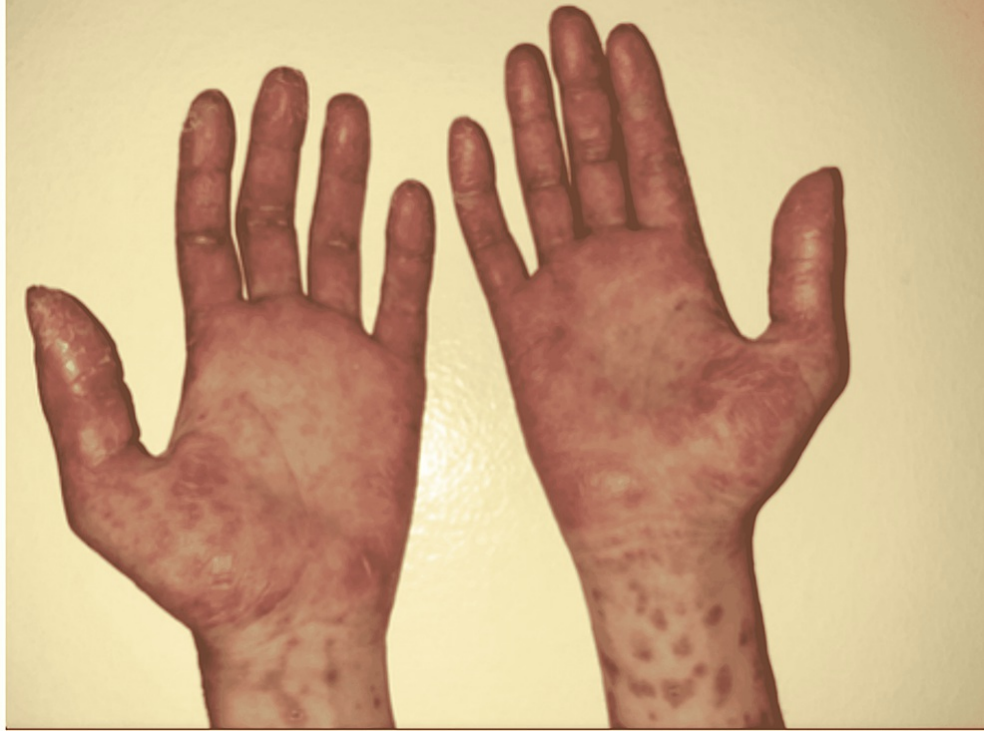
Cutaneous vasculitis located in the palmar regions of our patient.

The etiological assessment did not identify any infectious, toxic, or drug cause. The patient presented with lesions of cutaneous vasculitis at the palmoplantar regions (Figure 3), and oral and nasal ulcerations with polyarthritis of the hands.

The autoimmune assessment showed anti-nuclear antibodies greater than 1:1,280 with native anti-DNA antibodies at 210 UI/mL (N < 10), positive anti-SM Abs, and C3/C4 fractions consumed. Anti-cardiolipin, anti-beta2GP1, and lupus anticoagulant antibodies were negative. Endomyocardial biopsy was discussed but not performed after weighing risks and benefits for the patient in a multidisciplinary meeting. The diagnosis of lupus myopericarditis complicated by cardiogenic shock was made, in addition to renal involvement. In addition to the medical treatment of heart failure associated with pericardiocentesis for the pericardial effusion, a treatment was started with methylprednisolone at 1 g/day for three days and then prednisolone at 1 mg/kg/day, combined with mycophenolate mofetil at a rate of 2 g/day and hydroxychloroquine. On day 15, the left ventricular ejection fraction improved to 45-50%, and the patient showed good clinical improvement, and her heart failure and lupus signs improved.

## Discussion

Cardiac involvement in systemic lupus has a large spectrum (pericarditis, endocarditis, valvular lesions, myocarditis, cardiac arrhythmia, and conduction disturbance), but pericarditis constitutes the most frequent cardiac feature. Myocarditis remains rare but life-threatening with significant morbidity and mortality in 50% of cases, especially when it is present as an initial manifestation [5]. Few cases have presented with myocarditis complicated with cardiogenic shock associated with systemic lupus [2,4-7]. Pleural and pericardial effusion occur in a large number of patients with myocarditis, and this was the case of our patient too. This could be because myocarditis occurs during lupus mainly in the setting of myopericarditis [8].

The pathogenesis is thought to be in theory mediated by immune complex formation and complement activation [4,9]. Other contributing factors such as accelerated atherosclerosis, infections, antiphospholipid antibodies, and drugs such antimalarials were also described [9-10]. Surprisingly, a recent case-control study did not find any significant association of antiphospholipid antibodies with overt myocardial dysfunction in patients of systemic lupus [9].

It can be challenging to think of this condition when it is the first presentation of the disease, as was the very case of our young patient without any risk factors. Therefore, the search for extracardiac signs such as skin, joints, and kidney injuries can guide the etiological workup. Diagnostic workup must include a search for viral myocarditis, toxic or hypersensitivity-inducing drugs myocarditis, lupus myocarditis, other immune-mediated myocarditis (giant-cell myocarditis, or sarcoidosis, for example), and familial cardiomyopathy [7,10]. In that sense, patients with myocardial infarction and non-obstructive coronary arteries (MINOCA) represent an especially challenging population, especially on the etiological level, hence the difficulty to propose a proper therapy. A diagnosis of myocarditis is based on clinical, biological, radiological, and histological parameters. However, our patient did not undergo an endomyocardial biopsy, even if it is considered the gold standard for diagnosis of myocarditis [11]. Nowadays, cardiac magnetic resonance imaging is considered as a good non-invasive alternative in patients with suspected myocarditis [12], and it was the key for the diagnosis in our observation. In this direction, the current NSTEMI guidelines recommend cardiac magnetic resonance imaging in all patients with a first diagnosis of MINOCA, and this will facilitate the diagnosis of myocarditis in the future [10]. Patients suffering from myocarditis are mostly male and young adults [10], but when an autoimmune cause is involved, it occurs more in female patients, like in our case. Some recent studies also suggested 18F-FDG-PET/CT as a tool for the early diagnosis of myocarditis in systemic lupus [13].

The fact that our patient had negative cardiac enzymes (troponin was negative twice) rendered diagnosis even more difficult. It is reported that troponin positivity occurs in only 34% of cases and that is even true in early cases of autoimmune myocarditis [14,15]; therefore, we think that our patient has sought care belatedly.

There are no controlled studies or standard guidelines for treating lupus myocarditis [16]. In most cases, pulse corticosteroids followed by cyclophosphamide have been effective in lupus myocarditis management [8]. Other medications were used with various effects, such as azathioprine, cyclosporine, intravenous immunoglobulins, plasma exchange [8,17], and rituximab [18]. In few cases, mycophenolate mofetil was used as an induction drug in some cases with success [19-21], and significant improvements were noted in left ventricular ejection fraction with an overall cardiac recovery even in patients not treated with cyclophosphamide. To confirm the efficacy of mycophenolate mofetil in myocarditis, more data are needed. Given the rarity of this condition, controlled trials seem difficult to achieve and an international collaboration is much needed to collect cases from all over the academic world.

## Conclusions

The existence of myocarditis without obvious etiology of cardiogenic shock must make us look for systemic lupus in order to start an etiological treatment in addition to the cardiac treatment. Currently, there is no codified treatment for lupus myocarditis, but mycophenolate mofetil appears to be a promising treatment. This therapeutic effect must be confirmed by clinical trials, which remain difficult to conduct due to the rarity of this impairment. The importance of using effective medication is mandatory especially in a resource-limited country like ours, where the availability of cardiac transplantation is not a reality yet.

## References

[1] Moder KG, Miller TD, Tazelaar HD (1999). Cardiac involvement in systemic lupus erythematosus. Mayo Clin Proc.

[2] Appenzeller S, Pineau CA, Clarke AE (2011). Acute lupus myocarditis: Clinical features and outcome. Lupus.

[3] Wijetunga M, Rockson S (2002). Myocarditis in systemic lupus erythematosus. Am J Med.

[4] Mohanty B, Sunder A (2020). Lupus myocarditis-a rare case. J Family Med Prim Care.

[5] Ashrafi R, Garg P, McKay E, Gosney J, Chuah S, Davis G (2011). Aggressive cardiac involvement in systemic lupus erythematosus: a case report and a comprehensive literature review. Cardiol Res Pract.

[6] Durrance RJ, Movahedian M, Haile W, Teller K, Pinsker R (2019). Systemic lupus erythematosus presenting as myopericarditis with acute heart failure: a case report and literature review. Case Rep Rheumatol.

[7] Lee SY, Park JH, Shin DH (2021). Fatal myopericarditis in a patient with lupus erythematosus supported by extracorporeal membrane oxygenation: a case report. J Rheum Dis.

[8] Thomas G, Cohen Aubart F, Chiche L (2017). Lupus myocarditis: initial presentation and longterm outcomes in a multicentric series of 29 patients. J Rheumatol.

[9] Gawalkar AA, Bahl A, Ahluwalia J, Sood A, Sharma A, Sharma S, Dhir V (2020). Prevalence of antiphospholipid antibodies in patients with overt myocardial dysfunction in systemic lupus erythematosus. A case-control study. Lupus.

[10] Golpour A, Patriki D, Hanson PJ, McManus B, Heidecker B (2021). Epidemiological impact of myocarditis. J Clin Med.

[11] Bozkurt B, Colvin M, Cook J (2016). Current diagnostic and treatment strategies for specific dilated cardiomyopathies: a scientific statement from the American Heart Association. Circulation.

[12] Patriki D, Gresser E, Manka R, Emmert MY, Lüscher TF, Heidecker B (2018). Approximation of the incidence of myocarditis by systematic screening with cardiac magnetic resonance imaging. JACC Heart Fail.

[13] Perel-Winkler A, Bokhari S, Perez-Recio T, Zartoshti A, Askanase A, Geraldino-Pardilla L (2018). Myocarditis in systemic lupus erythematosus diagnosed by 18F-fluorodeoxyglucose positron emission tomography. Lupus Sci Med.

[14] Smith SC, Ladenson JH, Mason JW, Jaffe AS (1997). Elevations of cardiac troponin I associated with myocarditis. Experimental and clinical correlates. Circulation.

[15] Chattopadhyay P, Dhua D, Philips CA, Ghosh J (2011). A patient of lupus presenting with myocarditis and overlapping autoimmune hepatitis. Case Rep Rheumatol.

[16] Fanouriakis A, Tziolos N, Bertsias G, Boumpas DT (2021). Update οn the diagnosis and management of systemic lupus erythematosus. Ann Rheum Dis.

[17] Malhotra G, Chua S, Kodumuri V, Sivaraman S, Ramdass P (2016). Rare presentation of lupus myocarditis with acute heart failure-a case report. Am J Ther.

[18] Wang CR, Tsai YS, Li WT (2018). Lupus myocarditis receiving the rituximab therapy-a monocentric retrospective study. Clin Rheumatol.

[19] Al-Nokhatha SA, Khogali HI, Al Shehhi MA, Jassim IT (2019). Myocarditis as a lupus challenge: two case reports. J Med Case Rep.

[20] Marijanovich N, Halalau A (2018). Hemorrhagic tamponade as initial manifestation of systemic lupus with subsequent refractory and progressive lupus myocarditis resulting in cardiomyopathy and mitral regurgitation. Case Rep Rheumatol.

[21] Martorell EA, Hong C, Rust DW, Salomon RN, Krishnamani R, Patel AR, Kalish RA (2008). A 32-year-old woman with arthralgias and severe hypotension. Arthritis Rheum.

